# A comprehensive study on criteria of sustainable urban waste management system: using content analysis

**DOI:** 10.1038/s41598-023-49187-x

**Published:** 2023-12-18

**Authors:** Farhad Khosravani, Enayat Abbasi, Shahla Choobchian, Mahdi Jalili Ghazizade

**Affiliations:** 1 Department of Agricultural Extension and Education, Tarbiat Modares University, Tehran, Iran; 2 Department of Environmental Technologies, Shahid Beheshti University, Tehran, Iran

**Keywords:** Environmental sciences, Chemistry

## Abstract

The present study was developed to comprehensively analyze experts' views and content of documents focusing on goals and criteria of sustainable waste management system in Tehran, Iran. To this end, the suitable goals for sustainable waste management system in Tehran city were adopted from domestic, national and international documents. For this purpose, 27 national and international documents and 2 domestic documents related to waste management were selected and analyzed by using content analysis according to Gall, 1994. Further, in order to formulate goals in case of bottlenecks and challenges of waste management in Tehran, the focus group technique was used based on Stewart and Shamdasani, 2014. At this stage, 24 key experts in the field of waste management were interviewed in the form of 4 focus groups. Data collection were performed via audio recording and word-for-word implementation of conversations, taking notes and writing field notes. The data collection continued until reaching theoretical saturation. Next, content analysis and coding methods were used to analyze the data. Finally, the goals of waste management were divided into five general categories including: institutional (with emphasis on the integration and inclusion of the key elements of the urban waste management system), technical and infrastructural (with emphasis on the optimization of existing processes in the use of urban waste management technologies), environmental (with emphasis on minimizing the adverse health and environmental effects of the urban waste management system, economic (with emphasis on the economic and financial sustainability of the urban waste management system), and cultural-social (with emphasis on attracting the maximum participation of citizens and service recipients). The results clearly showed that sustainable waste management measures in Tehran should follow these five components in order to reduce the problems caused by unrealistic waste management and make sustainable use of basic, natural, financial and human resources.

## Introduction

**Figure 1:**
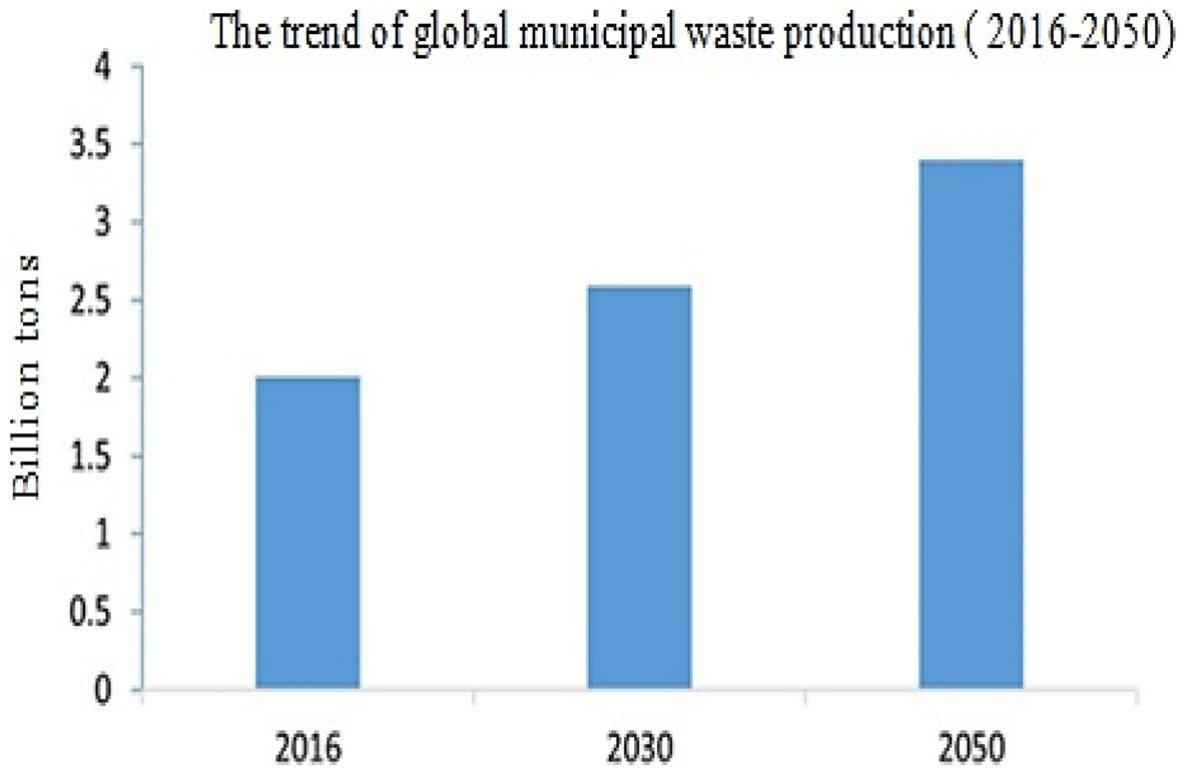
Global municipal waste generation trend (2016–2050), (in billion tons). Source^7^.

Last century evolutions along with the increased population growth have led to a new phenomenon of environmental destruction. One of the major environmental pollutants, which is considered as an integral part of human life, is waste and waste materials^1^. Currently, waste management is known an important and noticeable issue all over the world^2^. Waste management is considered as a major challenge all over the world, especially, cities with fast population growth in developing countries^3^. Poor collection and disposal of municipal solid waste leads to a variety of contaminants. In general, the accumulated waste in urban disposal centers causes the production of sewage and the cultivation of pathogenic organisms and insects. On the other hand, waste left in the seabed and erosion of coastal waste disposal sites lead to sea pollution and cause significant environmental damage. In this regard, it is predicted that if decisive steps are not taken to limit the amount of waste, more plastic waste will be observed in the oceans after 2050 than fish^4^. It is reported that the annual production of global municipal solid waste is about 2.01 billion tons, of which approximately 33% is not managed in a proper way^5,6^. It is projected that global waste production will reach 3.4 billion tons by 2050^5,7^ (Fig. 1).

Therefore, the need to dispose and process of municipal solid waste (MSW), especially in developing countries has become a serious issue^8,9^. Urban waste management mainly affects the standard of living of communities such as cleanliness, health and productivity^10,11^. Therefore, moving towards the optimal waste management with a perspective on sustainable development is one of the main goals of different societies in developed and developing countries^12,13^. The way of municipal waste management in developed countries is based on the maximum waste separation at source, recycling (due to its multiple financial and environmental benefits). For instance, in countries like Switzerland and Germany, 50–67% of food and dry waste is separated and recycled^14^. In addition, in United States, the percentage of waste separation and recycling has reached 56% from about 6% within a half of a century. Meanwhile, in Iran, it is reported that the at-source separation rate of food waste is less than 10%^15^; it is important to note that, the waste generation rate of Iranian people is expected to be 600 gr capita per day.

The ever-increasing growth of Iran's urban population along with the creation of new population centers, the lack and weakness of policy and evaluation of various urban activities based on the comprehensive and macro-national plan, are among the crisis-generating factors that damage the natural environment and the quality of health and health of humans, especially in city dwellers. On the other hand, many characteristics of urban waste in Iran, such as per capita waste production and the composition of its components, urban waste collection methods, various methods of waste disposal, are different from the methods commonly used in developed and industrialized countries and even some developing countries. Planners and authorities should focus seriously on these issue and act some measurements in a scientific and practical way in order to solve the problems for each element of waste management^16^.

In Iran, the lack of participation and correct management and clear regulations for collecting, disposing and recycling of more than 40 thousand tons of waste during the day (of which approximately 70% can be recycled and turned into compost and include thousands of tons of food waste) make situations to be indiscriminately dump or scattered around cities, deteriorating the health risks and also cause large economic losses^17^. The increase in population leads to increases in food consumption and ultimately increased generation rate of food waste. Tehran with a population of more than 10 million people generate about 7000 tons of waste per day, of which about 60% (i.e. 4200 tons) is organic and putrescible waste^16^. It can cause many problems including the spread of air pollution and environmental threats which are a warning for the health of the citizens and the environmental health of this metropolis^17^.

On the other hand, the development of the city of Tehran, along with the increase in population and changes in lifestyles, including consumption patterns, has created complications in the urban society, causing tremendous changes in the quantity and quality of waste. These changes and complexities have also led to numerous and diverse problems and dilemmas, such as problems in how to move and how to dispose of waste^18^.

Therefore, the facts and problems encountered in the waste management system of Tehran shows that most of the problems in this system are attributed to not paying attention to the goals of sustainable waste management. Generally, it can be argued that the goal of sustainable waste management is the sustainable use of resources in order to ensure the present and future well-being of humans and ecosystems. Waste management has provided the basis for conducting various researches. Abdoli^19^ investigated waste management in order to preserve nature and the urban environment. The authors concluded that one of the most acute environmental problems in Iran is the improper management of special industrial waste in urban areas. They stated that in order to preserve urban environment, it is necessary to enact a specialized law in the field of waste management.

In addition, Sorrentino et al.^20^ emphasized the importance of knowledge and awareness in household waste management. They claimed that strengthening the knowledge of waste management lead to improve the people's attitudes, leading to positive action in waste management. Furthermore, Zhang et al.^21^ investigated the effective factors in household waste separation behavior, and the results indicated that environmental knowledge and moral obligations both have a positive effect on environmental attitude, which can lead to the intended environmental behavior.

Lederer et al.^22^ surveyed the role of knowledge and awareness of stakeholders and local people in urban solid waste management in Basia, Uganda. The results indicated that in order to improve environmental conditions, stakeholders and community members should participate effectively in solid waste management along with government institutions.

Song et al.^23^ studied the environmental benefits, resources and energy through the improvement of urban waste management. The results showed that urban waste management has a great task to achieve the sustainability of a region. Because the upward trend of using fossil resources and the limitation and non-renewability of said resources along with the environmental pollution caused by them, has led to the direction of mankind towards the use of renewable resources and clean energy^23^.

There have been various studies on waste management in Tehran. For instance, in a study an analysis on the waste produced from 100 industrial units were conducted^24^. The results showed that 40.80% of the waste was transferred to the sanitary waste disposal site, 2.37% was buried in centers outside the control of the municipality, 2.1% was recycled and 7.1% was also burned uncontrolled. Damghani et al.^25^ focused on examining the quantity and quality of waste management in Tehran. The authors reported that the correct collection and management of hospital waste, need for personnel training in order to optimize waste collection and the participation of all public stakeholder institutions and the private sector are the challenges of waste management. Many studies emphasizes the provision of models for optimal sustainable management of urban waste, taking into account various economic, social and environmental dimensions. The models presented can be effective in providing prospects to managers in order to optimize the performance of the waste management system^26,27^.

A study examined the sustainability requirements of the waste management system from the perspective of the environment, economy and society with a focus on the location of waste management facilities. With the implementation of this model in Tehran, the potential for a reduction in fixed costs was calculated to be 12%, while a reduction in transmission costs of 22% and a promotion of system desirability were estimated at 17%^28^.

Fami et al.^29^ showed that each person enters the waste cycle on average about 27.6 kg of food a year. Food consumption management etiquette, economic power, and awareness and motivation directly affect food waste. Zand et al.^30^ showed that having an acceptable level of citizenship knowledge does not necessarily increase their participation in the waste separation scheme from the source, but it is recommended to implement immediate educational programs as well as adopt strict laws with applicability during the pandemic.

The results of the studies showed that the aim of sustainable waste management is to sustainably use basic, natural, financial and human resources to reform the consumption pattern and ensure the well-being of humans and ecosystems. On the other hand, waste management has never been explicitly mentioned in the sustainable development goals. With the knowledge that improving the waste management system as a goal can help achieving sustainable development goals in various dimensions, the need to extract the main concepts and axes of waste management and adapt it to sustainable development indicators and provide new, efficient and viable strategies in waste management is felt. Therefore, the present study, while studying external and internal documents as well as in-depth examination of the perspective of experts, has developed to provide strategies in the form of proposals while identifying the objectives of the sustainable waste management system. From this point of view, given that no research has been done on this topic, this research can help the Waste Management Organization of Tehran City in a successful management program in order to sustainable waste management. In other words, this article attempts to answer the fundamental question of what pivotal objectives should be considered in sustainable waste management based on subordinate documents.

To this end, present study was designed in three consecutive stages.

1. Determination the goals enacted in domestic and international documents;
2. Determination the goals enacted in the bottlenecks and challenges of waste management in Tehran based on the experts' point of view and
3. A comparative study on documents and experts' views in order to determine the implementation solutions to achieve the goals of sustainable waste management system in Tehran.

## Methodology

### Study area

**Figure 2:**
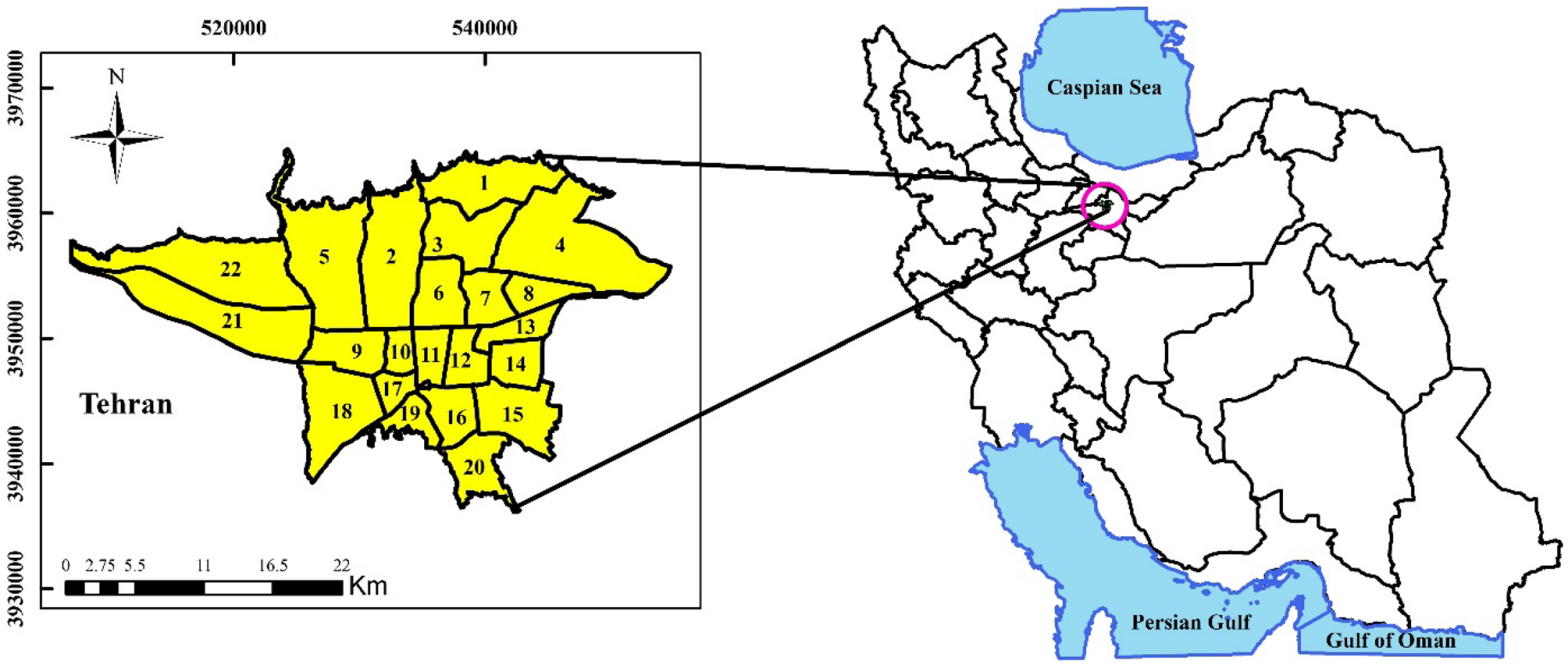
Geographical location of the study area.

The geographical area of the present study is the city of Tehran. Tehran, the capital of Iran, is the largest and most populous city with an area of 615 km^2^ and a population of about 9 million people. It is one of the largest cities in southwest Asia and the 21st largest city in the world. The city of Tehran in northern Iran, in the southern foothills of the Alborz mountain range, is located at a distance of 51° and 2 min east to 51° and 36 min east, approximately 50 km long and 35° and 34 min north to 35° and 50 min north to approximately 30 km wide. The elevation of the city reaches 2000 m in the highest parts of the North and 1050 m above sea level in the southernmost point. Tehran leads from the north to mountainous areas and from the south to desert areas, resulting in a different climate in the North and South. The northern regions have cold and dry climates and the southern regions have hot and dry climates. Tehran has 22 districts, 123 districts and 354 neighborhoods (the location of the city of Tehran and the map of Iran can be seen in Fig. 2)^31^.

In this qualitative study, content analysis method, review of documents and expert panel were employed in order to extract the goals of sustainable waste management.

### Monitoring the international, national and domestic documents

To this end, at first domestic, national and international documents focusing on sustainable goals of waste management were surveyed. In this section, 27 national and international documents and 2 domestic documents related to waste management were selected through media scanning and internet search (Tables 1 and 2). It is noteworthy that in the review of foreign experiences in the field of waste management documents of different cities in the world, the priority in the reviews has been access to the documents of their comprehensive waste management plans, and therefore, although an attempt has been made to select cities with a similar situation to Tehran; however, due to the lack of governance of the strategic planning approach on the process of preparing comprehensive waste management plans in Asian cities and the Middle East region, the frequency of cities in advanced and developed countries such as Europe and America is higher in this survey.

**Table 1:** Examining national and international waste management documents.

No.	Country	City	Document	Year	Refs.
1	Jordan	–	SWIM and Horizon 2020 Support Mechanism	2015	^32^
2	Myanmar	Mandalay	City Waste Management Strategy and Action Plan for Mandalay	2016	^33^
3	Tasmania	Hobart	Waste Management Strategy (2015–2030)	2015	^34^
4	Great Britain	London	The Mayor’s Draft Municipal Waste Management Strategy London’s Wasted Resource	2010	^35^
5			Waste Strategy (2013–2020)	2014	^36^
6		West Berkshire	A Municipal Waste Management Strategy for West Berkshire Council	2002	^37^
7	USA	Phoenix	Solid Waste Strategic Plan Fiscal Years (2016–2021)	2015	^38^
8		Marathon City from the state of Wisconsin	Marathon County Solid Waste Management Board Strategic Plan	2014	^39^
9		Pasadena	Zero Waste Strategic Plan Solid Waste (2038)	2014	^40^
10		State of Rhode Island	Rhode Island Comprehensive Solid Waste Management Plan	2015	^41^
11		Lincoln and Lancaster	Solid Waste Management Plan for Lincoln and Lancaster County (2040)	2013	^42^
12		Enoka, Carver, Dakota, Hennepin, Rumsey, Scott and Washington counties	Metropolitan Solid Waste Management Policy Plan (2016–2036)	2016	^43^
13		Massachusetts	Massachusetts 2010–2020 solid waste master plan Pathway to Zero Waste	2013	^44^
14		Ramsey County, Minnesota	Solid Waste Management Master Plan (2018–2038)	2018	^45^
15	Kenya	–	Health Care Waste Management Strategic Plan (2015–2020)	2015	^46^
16		–	The National Solid Waste Management Strategy	2015	^47^
17		Nairobi	Integrated Solid Waste Management Plan For the City of Nairobi, Kenya (2010–2020)	2010	^48^
18	Sierra Leone	–	Integrated National Waste Management Strategic Plan (2012–2016)	2011	^49^
19	Canada	Toronto	Zero Waste (2040)	2016	^50^
20		Vancouver	Preparation of Regional Waste Management Plans and Strategic Environmental Assessments for East and North-East region	2018	^51^
21	Eastern and northeastern regions of Europe	–	Preparation of Regional Waste Management Plans and Strategic Environmental Assessments for East and North-East region	2014	^52^
22	India	Pune	Solid Waste Management Strategy Plan (2017–2025) (Pune Municipal Corporation)	2017	^53^
23	Australia	Mitcham	Waste Management Strategy (2016–2021)	2016	^54^
24		Macedon Ranges Shire Council	Waste Management Strategy (2015–2020)	2014	^55^
25		Busselton	Strategic Waste Management Plan (2016–2020)	2014	^56^
26		Melbourne	The City of Melbourne Waste Management Strategy	2005	^57^
27	United Nation	United Nations Environment Program	Developing Integrated Solid Waste Management Plan Training Manual Volume 4: ISWM Plan	2009	^58^

**Table 2:** Examining domestic waste management documents.

No.	Country	City	Document	Year	Refs.
1	Iran	Shiraz	Comprehensive waste management plan for Shiraz city (with a horizon of 1400)	2009	^59^
2		Isfahan	Strategic plan for Isfahan Municipal Waste Management Organization (1400)	2016	^60^

The researcher-made forms were used to collect the data. This section of study was performed based on document content analysis according to method by Gall^61^ in five stages. In the first stage, the mentioned documents were carefully studied and a set of propositions contributing on key concepts in the field of waste management were extracted.

In the second stage, the appropriate categorization method was selected. The authors tried to make each category representative of a separate variable and objectives of present research. In the third step, each collected proposition was marked to be placed in each category.

In other words, each statement propositions extracted from the documents was examined to determine whether the phenomenon described in each statement propositions fits into one of the desired categories or not. In the fourth step, categorization was done with open and axial coding. The purpose of implementing axial coding was to create relationships between the categories produced in the open coding stage. Finally, in the final stage, the data were processed and the results were interpreted.

### Formulation the sustainable goals based on bottlenecks and challenges in waste management

In the second step, a focus group technique was used in order to formulate goals derived from the bottlenecks and challenges of waste management in Tehran. The focus group technique was performed according to Stewart and Shamdasani^62^ in eight stages: (1) defining the problem (what are the bottlenecks and challenges of waste management in Tehran?), (2) identifying the sampling frame, (3) choosing a facilitator or coordinator, (4) Inviting participants, (5) compilation of interview guidelines, (6) focus group implementation, (7) data collection and analysis and (8) report preparation.

The members of the focal group in this step included 24 key experts involved in different elements of waste management: managers and experts in Tehran Waste Management Organization, Tehran Municipality Districts, Tehran City Environmental and Urban Services Deputy, Tehran City Planning and Studies Center and Tehran Municipality Environment and Sustainable Development Department, which were selected using the snowball sampling method. At this stage, the interviews were conducted in the form of four focus groups.

Data collection and information in this part of the research were performed via audio recording and word-for-word implementation of conversations, along with note-taking and field notes. The average time for each session was 80 min and the number of participants in each focus group was between 6 and 8 people, and data collection continued until theoretical saturation. In this stage, method of content analysis and coding (open and axial) was used in order to analyze the results.

### Consent to participate

The authors agree to participate in this research study.

## Results

### A review on national, international and domestic documents of urban waste management

In the first stage of five-step document content analysis approach according to Gall 1994. A set of semantic units and key concepts of waste management were extracted. These concepts were summarized according to their importance and the amount of emphasis on them. In fact, at this stage, all the propositions with characteristics to be able to deduce a set of key concepts in the field of waste management were extracted (Tables 3 and 4).

**Table 3:** A review on national and international waste management documents.

Country	City	Extracting the key concepts of waste management	Frequency
Jordan	–	Affordable Efficient Comfortable—environmentally and society friendly	4
Myanmar	Mandalay	Clear Green Healthy	3
Tasmania	Hobart	Completion of waste burial operations Reducing the amount of waste to zero	2
Great Britain	London (2010)	Leading city in terms of urban waste management Reducing the effect of urban waste on climate change Fully exploiting the financial value of urban waste through the use of creative techniques and technologies Reducing the amount of urban waste Increasing compost recycling and performance Energy production from waste	6
Great Britain	London (2010)	Reuse of waste Waste recycling Waste reduction Waste management	4
USA	Phoenix	Sustainability Optimal management of resources	2
Kenya	–	A healthy and safe environment Free from diseases, hospital wastes, risks and environmental pollution	2
Sierra Leone	–	Provider of efficient and high-quality services Accessible Justly Affordable	4
Canada	Toronto	Reducing the amount of production waste Reuse of resources and facilities Recovery and recovery of resources and its return to the economic cycle User-friendly waste management system Maintaining balance between society, environment and financial stability A safe, clean, beautiful and healthy city	6
Kenya	–	Sustainable and efficient waste management system	1
Eastern and northeastern regions of Europe	–	Sustainable waste management Resource recovery Integrated waste management system	3
India	Pune	A clean city; Efficient waste management	2
U.S.A	Marathon city from the state of Wisconsin	Integrated waste management system Strengthening economic development Protection of the environment and public health of society	3
Australia	Mitcham	Reducing the amount of production waste Waste recycling Maintaining health	3
Great Britain	West Berkshire	Reducing the amount of production waste Waste recycling Maintaining health	3
Australia	Macedon Ranges Shire Council	Eliminating or reducing waste Increasing recycling and recovery of material resources Improving productivity and environmental protection	3
Kenya	Nairobi	Healthy waste management system Safe waste management system Secure waste management system Sustainable waste management system Global waste management system	5
Australia	Busselton	Strengthening the power of the local economy and activities with added value Development of waste services and infrastructures Avoiding the generation of waste, reducing and recycling it, and observing appropriate waste disposal standards Informing, training and community participation in waste management	4
USA	Pasadena	A city without waste	1
Canada	Vancouver	Society without waste Sustainable use of resources Healthy economy Rich, vibrant and inclusive neighbourhoods Equal opportunities	5
U.S.A	State of Rhode Island	Reducing the amount of waste Implementation of waste fee payment	2
U.S.A	Lincoln and Lancaster	Maintaining and promoting the health, safety, and welfare of society Promoting ideals and values Meeting the needs of the current generation without jeopardizing the ability of future generations to meet their needs	3
Australia	Melbourne	Continuous improvement of recycling process and sustainable consumption Individual initiatives and creativity in different and optimal use of resources Production and supply of environmentally friendly products and services High standards in waste minimization and recycling Promoting local insight and participation in recycling and sustainable consumption Compliance with sustainability considerations in packaging, production and service provision by companies and commercial businesses The challenge of improving waste reduction methods and optimal use of resources by employees and managers of companies and economic enterprises Reducing the amount of production waste Providing innovative and efficient waste collection services Providing a wide range of environmentally friendly products and goods Promoting and implementing commercial activities, events and cultural-artistic events compatible with the environment The pleasure of feeling and experiencing sustainability in events and buying goods and products Design and green architecture	13
U.S.A	Enoka, Carver, Dakota, Hennepin, Rumsey, Scott and Washington counties	Minimizing the amount of waste Prevention of contamination Reduction of greenhouse gas emissions Development of resources in line with revitalization of the local economy Integrated waste management system Reuse of resources Resource recovery	7
U.S.A	Massachusetts	Reducing the amount of waste Reducing pollution caused by waste Minimal disposal of waste Use of environmentally friendly waste transportation equipment	4
U.S.A	Ramsey County, Minnesota	Taking advantage of new technology and technologies Enjoying the economic benefits of waste Increasing the amount of recycling Reducing the amount of waste; Protecting the interests of toll payers and the environment of citizens	5
United Nation	United Nations Environment Program	Reducing the amount of waste Improving resource management Protecting public health and safety of society and the environment	3

**Table 4:** A review on domestic waste management documents.

Country	City	Extracting the key concepts of waste management	Frequency
Iran	Shiraz	Developed in the field of waste management Pioneer and achieved the first position of services in the field of waste management at the country and regional level Reaching the location of clean city Regular and integrated policy in planning Participation and attraction of private sector capital Responsible and effective cooperation and participation of service recipients Capable manpower in the field of waste management Existing cultural technologies in the field of waste management Responsible human force with working conscience, discipline, cooperative and committed spirit Constructive and effective interaction	10
	Isfahan	Valid in normal urban waste management Excellence in controlling and reducing the vulnerability of the environment Protector of natural resources and health of the urban environment Development of clean city	4

After extraction concepts from the documents, the key concepts were divided into five categories: (1) Institutional, (2) Technical and infrastructural, (3) Economic, (4) Environmental and (5) Socio-cultural categories. These categories include all extracted concepts. Next, each concept were placed in each corresponding categories. In the fourth stage, the extracted concepts were classified based on similarity in the form of institutional, technical and infrastructural, economic, environmental and socio-cultural categories (Table 5). Finally, in the fifth stage, based on the results of open and axial coding, the categories were systematically processed and analyzed in relation to other categories. Table 5 summarizes the different categories and key concepts related to sustainable goals for solid waste management.

**Table 5:** Different categories of sustainable goals for solid waste management and their corresponding key concepts.

No.	Categories	Inferential concepts and goals	Frequency
1	Institutional	Integrated urban waste management	5
		Promotion of inter-departmental and inter-organizational coordination	4
		Planning and emergency management of waste in order to guarantee the mobilization of all facilities when natural and unexpected events occur	5
		Achieving the legal requirements for municipal solid waste management	2
2	Technical and infrastructural	Improving the final waste disposal and landfill	5
		Implementation of the best urban waste management measures	6
		Optimizing the waste management process	7
		Improving the material recycling cycle	4
		Extraction and production of clean energy	4
		Optimum use of mandatory elements of the waste management system, such as reducing the use of resources, recycling and composting to reduce the amount of landfill waste	3
		Composting and recycling all compostable and recyclable materials	3
		Increasing source separation of household hazardous waste	2
		Setting up the Materials and Resources Innovation Center	1
		Easy and up-to-date waste recycling process	1
		Guarantee the quality and continuity of waste collection services to all applicants	3
		Creating and launching a cost-effective, efficient, applicable waste management system that provides quality services and is friendly to the environment and society	5
		Ensuring sustainable service delivery through review, monitoring, improvement and innovation in it	4
		Design, development and implementation of all facilities and equipment of the waste management system with an emphasis on sustainability requirements	3
3	Economic	Minimizing waste costs and risks for society	10
		Increasing the economic benefits of waste through better use of materials and material resources	8
		Helping to launch and expand sustainable consumption markets to support the return of discarded materials to the economic cycle	8
		Support the delivery of local and indigenous initiatives to promote local employment and economic growth	5
		Revision of the framework and structure of waste fee payment	5
		Maximum reimbursement of expenses	4
		Empowering citizens and economic activists to benefit from the economic benefits of waste management	1
4	Environmental	Encouragement to reduce the amount of waste and reuse it as much as possible	6
		Reducing the amount of waste	7
		Avoiding waste production and minimizing it	8
		Removing toxic and dangerous substances from the municipal waste management cycle	4
		Ensuring optimal final disposal of unusable waste	6
		Proper collection and disposal of household hazardous waste	4
		Proper collection and disposal of industrial waste and infectious waste	4
		Controlling the hazardous nature of waste (special waste)	4
		Reducing the environmental effects caused by the production and disposal of waste	4
		Managing and reducing the effects of waste on the health and safety of society, and the environment	3
		Maximizing the conduction and disposal of liquid waste	2
		Reduction of greenhouse gas emissions resulting from the municipal solid waste cycle	2
5	Sociocultural	Increasing public participation	6
		Promoting public education	7
		Capacity building, awareness and gaining public support	7
		Modification of consumerist behavioral patterns	5
		Community empowerment and participation in avoiding waste production and recycling	1
		Expansion of educational programs in schools	1

### Categorization of objectives derived from the bottlenecks and challenges of waste management in Tehran in view point of experts

In the second stage of the research, goals derived from the bottlenecks and challenges of waste management in Tehran were extracted from the perspective of 24 key experts involved in waste management using focus group technique. The analysis of the results obtained from the interviews were performed using the content analysis method. Of note, the interview was performed in the form of four focus groups.

To this end, after extracting key concepts from the recorded content, appropriate categories were selected. In addition, due to the same nature of the goals of this stage with the previous one, in this part of the research, institutional, technical and infrastructure, economic, environmental and Socio-cultural were defined for different key concepts of sustainable goals in solid waste management (See Table 6).

**Table 6:** Different categories of sustainable goals derived from bottlenecks and challenges in solid waste management in Tehran and their corresponding key concepts.

No.	Categories	Bottlenecks and inferential challenges	Frequency
1	Institutional	Weakness in the central plan and specialization in the waste management system	8
		Weakness in the organization's management and outsourcing model, as well as how to evaluate and provide contractors' services	7
		Lack of integration of monitoring in the waste management cycle	7
		The necessity of reforming the organizational structure of waste management	5
		The organization's focus on executive actions and tasks instead of policy, planning and monitoring	5
		Lack of a specific mechanism to organize informal agents active in the field of dry waste	4
		The necessity of organizing and interacting with centers and social institutions active in the field of waste management	3
		Conflict and functional interference in the description of the duties of the main elements	3
		The number of contractors in the waste collection sector	2
		Existence of a legal vacuum in dealing with garbage collectors	2
2	Technical and Infrastructural	Weakness in providing the necessary infrastructure, personnel training and capacity building to use new technologies	16
		Absence of a central and integrated waste data recording system	15
		Low efficiency of the waste collection system and its high frequency	14
		Absence of an integrated system for recording information on reservoirs and the time and route of waste collection	10
		The necessity of including international standards and regulations in waste management activities	10
		Weakness in updating waste processing technology	8
		Low level of separation from the source of waste	8
		Lack of infrastructure and related facilities and services	8
		Improper storage system to increase separation from the origin	4
		low-quality compost production due to impurity and non-uniform production process	2
3	Economic	Weakness in financial transparency and sustainability of revenue sources of waste management organization	10
		Spending most of the waste management costs on the waste collection stage	6
		Inability to receive the price of waste management services	6
		Lack of integration in providing and spending system revenues	5
4	Environmental	High percentage of urban waste burial	17
		Higher level of waste production compared to the global average	13
		The necessity of measuring and monitoring the health, safety and environment of the waste management system	13
		The rising course of dry waste production	11
		The existence of adverse health and environmental effects in different parts of waste management	9
		Minimal use of protective equipment by garbage collectors and occurrence of health effects	7
5	Sociocultural	Low level of solidarity and social participation	19
		Low level of public knowledge in the field of waste management	15
		Lack of efficient participation of citizens in the matter of separating dry waste	15
		The spread of garbage collection and the formation of a large and powerful informal sector	11
		Activity of working children in the informal sector	10

### A comparative comparison derived from documents and experts' views in order to determine the implementation solutions to achieve the goals of the sustainable waste management system in Tehran

Finally, by adapting the bottlenecks and challenges of waste management in Tehran from the viewpoint of experts (existing situation) with the requirements of waste management taken from international, national and documents (desired situation), feasible and applicable solutions were presented in order to achieve the five goals of Tehran's waste management system (See Table 7).

**Table 7:** Comparative comparison of documents and experts' views in order to determine the implementation solutions to achieve the goals of the sustainable waste management system.

Objectives	Bottlenecks and challenges of waste management in the city of Tehran from the point of view of experts (Current situation)	Waste management requirements, taken from external and internal documents (desired situation)	Executive solutions to achieve the goals of sustainable waste management system in Tehran
Institutional	Weakness in program orientation and specialization in the waste management system Weakness in the organization's management and outsourcing model, as well as how to evaluate and provide contractors' services lack of integration of monitoring in the waste management cycle The necessity of reforming the organizational structure of waste management The organization's focus on executive actions and tasks instead of policy, planning and monitoring Lack of a specific mechanism to organize informal agents active in the field of dry waste The need to organize and interact with centers and social institutions active in the field of waste management Conflict and functional interference in the description of the duties of the main elements The number of contractors in the waste collection sector Existence of a legal vacuum in dealing with garbage collectors	Integrated urban waste management Promotion of inter-departmental and inter-organizational coordination Planning and emergency management of waste in order to guarantee the mobilization of all facilities when natural and unexpected events occur Achieving the legal requirements of municipal solid waste management	Defining the duties and mechanism of participation and interaction with all stakeholders and stakeholders of the waste management system Modifying the structure and relationships within and outside the system of the waste management organization of Tehran based on the approach of institutional agility and capacity building Strengthening the role and position of policy and decision-making of the waste management organization in the waste management system of Tehran
Technical and infrastructural	Weakness in providing the necessary infrastructure, personnel training and capacity building to use new technologies Absence of a central and integrated waste data recording system Low efficiency of the waste collection system and its high frequency Absence of an integrated system for recording information on reservoirs and the time and route of waste collection The necessity of consideration the international standards and regulations in waste management activities Weakness in updating waste processing technology Low level of separation from the source of waste Lack of infrastructure and related facilities and services Improper storage system to increase separation from the origin Low-quality compost production due to impurity and non-uniform production process	Improving the final waste disposal and burial system Implementation of the best urban waste management measures Optimizing the waste management process Improvement of material recycling cycle Extraction and production of clean energy Optimum use of elements of the waste management system, such as reducing the use of resources, recycling and composting to reduce the amount of landfill waste Composting and recycling of all compostable and recyclable materials Increasing separation from the source of household hazardous waste Setting up the Materials and Resources Innovation Center Easy and up-to-date waste recycling process Guarantee the quality and continuity of waste collection services to all applicants Creating and launching a cost-effective, efficient, applicable waste management system which provides quality services and is friendly to the environment and society Ensuring sustainable service delivery through review, monitoring, improvement and innovation in it Design, development and implementation of all facilities and equipment of the waste management system with an emphasis on sustainability requirements	Capacity building and empowerment of the waste management system of Tehran city in line with the application and creation of new innovations and technologies Continuous collection, classification and integration of information and descriptive and spatial data of waste management of Tehran city Developing the use of current knowledge and technologies during waste management operations, Expanding the use of modern physical infrastructure (facilities and equipment) in accordance with Tehran's requirements and environmental conditions
Economic	Weakness in financial transparency and sustainability of revenue sources of waste management organization Spending most of the waste management costs on the waste collection stage Inability to receive the price of waste management services Lack of integration in providing and spending system revenues	Minimizing waste costs and risks for society Increasing the economic benefits of waste through better use of materials and material resources Helping to launch and expand sustainable consumption markets to support the return of discarded materials to the economic cycle Support the delivery of local and indigenous initiatives to promote local employment and economic growth Revision of the framework and structure of waste fee payment Maximum reimbursement of expenses Empowering citizens and economic activists to benefit from the economic benefits of waste management	Integration, clarification and optimization of waste management system costs
Environmental	High percentage of urban waste burial Higher level of waste production compared to the global average The necessity of measuring and monitoring the health, safety and environment of the waste management system The rising course of dry waste production The existence of adverse health and environmental effects in different parts of waste management Minimal use of protective equipment by garbage collectors and occurrence of health effects	Encouragement to reduce the amount of waste and reuse it as much as possible Reducing the amount of waste Avoiding waste production and minimizing it Removing toxic and dangerous substances from the municipal waste management cycle Ensuring optimal final disposal of unusable waste Proper collection and disposal of household hazardous waste Proper collection and disposal of industrial waste and infectious waste Controlling the hazardous nature of waste (special waste) Reducing the environmental effects caused by the production and disposal of waste Managing and reducing the effects of waste on the health and safety of society, and the environment Maximizing the conduction and disposal of liquid waste Reduction of greenhouse gas emissions resulting from the municipal solid waste cycle	Minimizing the harmful health and environmental effects of the operation of the waste management system in Tehran
Sociocultural	low level of solidarity and social participation; Low level of public knowledge in the field of waste management Lack of efficient participation of citizens in the matter of separating dry waste The spread of garbage collection and the formation of a large and powerful informal sector Activity of working children in the informal sector	Increasing public participation promoting public education Capacity building, awareness and gaining public support modification of consumerist behavioral patterns Community empowerment and participation in avoiding waste production and recycling Expansion the educational programs in schools	Attracting the maximum participation and cooperation of citizens in the waste management system process Minimizing the socio-cultural effects of the waste management system

## Discussion

In this research, the content analysis of national, international and domestic documents and the opinion of experts revealed that one important sources of extracting and formulating the goals of the waste management system is the goals enacted based on the concept of "sustainable development". Despite the fact that waste management is not explicitly mentioned in the Sustainable Development Goals, improving the waste management systems as a goal can help achieve many of those goals.

Currently, many countries have enacted sustainable waste management as a priority in their agenda according to 2030 document; they have enacted the decisions for sustainable solid waste management in upcoming years based on this document^63^. In this regard, this research is in line with goal No: 11 of 2030 document, called "creating inclusive, safe, resilient and sustainable cities and towns" and sub-set of micro goal number 6, reduction of adverse environmental effects in cities, including management municipal waste is relevant.

**Figure 3:**
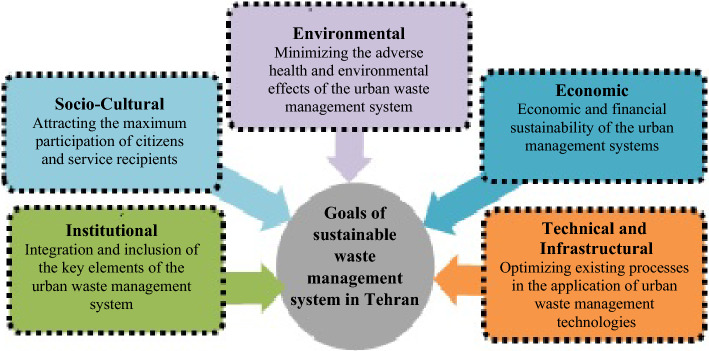
Goals of sustainable waste management system in Tehran.

Based on the results obtained from the present research and aligned with sustainable development goals, five general goals of sustainable waste management were identified (Fig. 3). In the following, each of these goals are discussed.

### Institutional goals

Institutional goals (with emphasis on the integration and inclusion of the key elements of the urban waste management system) is one of the extracted goals. Key concepts derived from documents focusing on sustainable goals of waste management (documents numbers 4, 5, 6, 8, 9, 10, 11, 12, 13, 17, 20, 23, 24, 27, 28, 29 in Tables 1 and 2) indicates that integrated urban waste management, planning and emergency management of waste and inter-departmental and inter-organizational coordination as well as achieving the legal mechanisms of urban solid waste management are considered as the requirements of this goal. Therefore, defining the duties and mechanism of participation and interaction with all stakeholders of the waste management system, reforming the structure and intra- and extra-systemic relations of the waste management organization of Tehran based on the approach of institutional agility and capacity building, strengthening the role and position of policy and decision making of the waste management organization are important parameters in the waste management system of Tehran city. In previous studies, institutional goals have been mentioned as one of the goals of sustainable urban waste management^19,64,65^. Creating legal, technical and executive frameworks in order to reduce the amount of production and expected to increase the separation, processing and recycling of waste, and the formulation and revision of rules, guidelines, guidelines and by-laws for waste management will pave the way for the realization of institutional goals.

### Technical and infrastructural goals

Technical and infrastructural goals (with emphasis on optimizing existing processes in the application of urban waste management technologies) is another goal of sustainable urban waste management. The results obtained from document review (documents numbers 1, 4, 5, 8, 9, 11, 14, 15, 16, 18, 19, 21, 23, 24, 25, 26, 27 in Table 1) showed the improvement the final disposal and waste burial system, optimization the waste management process, improvement the material recycling cycle, extraction and production of clean energy, optimal use of elements of the waste management system such as reducing the use of resources, recycling and composting to reduce the amount of landfill waste, increasing source-separation of hazardous waste, design, development and implementation of all facilities and equipment of the waste management system with an emphasis on sustainability requirements are the most important issues imposed in order to achieve technical and infrastructural goal. Expansion the modern physical infrastructure (facilities and equipment) according to Tehran's requirements and environmental conditions, gradual collection of 1100-L tanks available in the city and installation of smart tanks, design and implementation of information system and decision support for waste management and creation of necessary physical infrastructure in line with collection, registration, analyzing and disseminating of data and information on waste flow and developing the use of current knowledge and technologies during waste management operations are the proposed elements to achieve Technical and infrastructural goal. The findings related to this section are consistent with similar previous studies^66,67^.

### Economic goals

The economic goals (with emphasis on the economic and financial sustainability of the urban waste management system) is the third objective of sustainable urban waste management. The results (analysis of documents No. 1, 4, 8, 13, 18, 20, 21, 24, 26 in Table 1) showed a weakness in financial transparency, spending maximum costs on the collection stage, inability to receive the price of services and lack of integrity in providing and spending system revenues is one of the economic challenges of waste management in Tehran. Increasing the economic benefits of waste through better use of materials and resources, helping to launch and expand markets to support the return of discarded materials to the economic cycle, reviewing the framework and structure of waste fee payments, designing and implementing a Pay-for-Service (PAYT) system are the ways to achieve economic objective. In previous researches^66,68^ the economic and financial sustainability of sustainable urban waste management has been considered.

### Environmental goals

The environmental goals (with emphasis on minimizing the adverse health and environmental effects of the municipal waste management system) is another sustainable urban waste management^21,23^. Minimizing the harmful health and environmental effects related to operation of the waste management system in Tehran should be considered as the most important principle (documents Nos. 1, 2, 3, 4, 6, 7, 9, 12, 13, 15, 16, 20, 22, 23, 24, 25, 26, 27, 29 in Tables 1 and 2). Standardization the current processes and methods of waste management system operation, and reduction and control of leachate emission in roads and urban environment are proposed parameters to achieve environmental objectives.

### Socio-cultural goals

Socio-cultural goals (with emphasis on attracting the maximum participation of citizens and service recipients) is another influential goal in the sustainable management of urban waste^20,68^. The results obtained from investigation the socio-cultural goal in waste management of Tehran showed that the maximum participation of citizens and the reduction of negative socio-cultural effects in the process of the waste management system will expected to increase public participation, promote public education, modify consumerist behavior patterns, and empower citizens to avoid the waste production and improve the recycling of waste and the expansion of educational programs in schools (documents numbers 3, 4, 5, 14, 18, 22, 23, 28 in Tables 1 and 2). Creating an incentive package expected to increase the separation of waste from the source of residential units, improving the waste source separation in non-residential units, requiring major waste producing units such as fruit and vegetable fields, fruit shops, hotels, etc. to separate more waste at the source, revising the education system based on effective and innovative methods with integrated learning approach and focusing on group training and providing waste management training to schools, NGOs and other target groups are the main social and cultural measures and suggestions in order to achieve sustainable urban waste management.

## Conclusion

This research identified five general goals of sustainable waste management that are aligned with the goals of sustainable development. In fact, five dimensions include: institutional goal (with emphasis on the integration and inclusion of key elements of the urban waste management system), technical and infrastructural goal (with emphasis on optimizing existing processes in the use of urban waste management technologies), environmental goal (with emphasis on minimizing adverse health and environmental effects of the urban waste management system), the economic goal (with emphasis on the economic and financial sustainability of the urban waste management system) and the cultural-social goal (with emphasis on attracting the maximum participation of citizens and service recipients) were determined as the main effective goals in sustainable waste management.

It should be mentioned that in this research, due to the impossibility of reviewing all national and international and domestic waste management documents, the available documents were studied with a strategic planning approach, which is one of the limitations of the research.

Another limitation of the research is that there is absolutely little data to validate and verify the analysis and results obtained. Therefore, if the data is available, the authors can sort their findings on the "5 general objectives of sustainable waste management" in a lesser way.

Finally, according to the goals achieved in this research as the five main goals in sustainable waste management of Tehran city, it is suggested that the waste management strategies and policies of Tehran city can be considered at further research in order to achieve the mentioned goals.

## Data Availability

All data generated or analyzed during this study are included in this published article.
